# Psychiatric morbidity among patients on treatment for tuberculosis at a tertiary referral hospital in Western Kenya

**DOI:** 10.1371/journal.pone.0302744

**Published:** 2024-05-15

**Authors:** Robina Kerubo Momanyi, Edith Kamaru Kwobah, Philip Owiti, Henry Nyamogoba, Lukoye Atwoli

**Affiliations:** 1 Department of Mental Health and Behavioural Sciences, School of Medicine Moi University, Kenya; 2 Department of Mental Health, Moi Teaching and Referral Hospital, Kenya; 3 National Tuberculosis Program, Ministry of Health, Kenya; 4 Department of Medical Microbiology and Parasitology, School of Medicine Moi University, Kenya; 5 Department of Medicine, Medical College East Africa, Aga Khan University, Kenya; 6 Brain and Mind Institute Aga Khan University, Kenya; Médecins Sans Frontières (MSF), SOUTH AFRICA

## Abstract

**Background:**

Mental disorders account for nine percent of the overall global burden of disease and are among the top ten leading causes of disability. Mental illness and tuberculosis share risk factors including poverty, overcrowding, stigma, poor nutrition, substance use and retro-viral disease co-infection. Presence of mental illness in tuberculosis delays health-seeking, affects drug adherence, increases cost of treatment, prolongs disease duration, lowers quality of life, and increases mortality. Early diagnosis, linkage, and treatment of psychiatric morbidity among patients with tuberculosis would improve outcomes for both. This study thus aimed to determine the prevalence and factors associated with psychiatric morbidity among patients on treatment for tuberculosis at a low- middle- income country.

**Methods:**

A cross-sectional study carried out at the tuberculosis clinic at Moi Teaching and Referral Hospital (MTRH), Eldoret, Kenya. 367 participants on TB treatment were interviewed using Mini-International Neuropsychiatric Interview (MINI) tool. The key outcome was presence of psychiatric illness. Pearson’s Chi-square and logistic regression were used to assess relationships at bivariate and multivariate level respectively.

**Results:**

Majority of the respondents were male (61.3%) and overall median age was 33 years. About half of participants (48.5%) had at least one psychiatric illness. Common disorders were alcohol use disorder (30.3%), depression (23.4%), substance use disorder (12.8%) and suicidality (8.2%). Odds of ‘any psychiatric illness’ were increased by being male (aOR = 1.92; *P* = 0.04), being separated or divorced (aOR = 6.86; *P* = 0.002), using alcohol (aOR = 3.2; *P*<0.001), having been previously treated for tuberculosis (aOR = 2.76; *P* = 0.01), having other medical comorbidities (aOR = 4.2; P = 0.004) and family history of mental illness (aOR = 2.4; *P* = 0.049).

**Conclusion:**

Almost half of the patients on treatment for tuberculosis had at least one psychiatric illness. Introduction of protocols for screening for mental illness and integration of mental health services with tuberculosis care would aid prompt diagnosis, referral, and quality of care.

## Introduction

Mental disorders account for nine percent of the overall global burden of disease and are listed among the top ten leading causes of disability. Tuberculosis (TB) is the second leading cause of death from a single infectious disease with an estimated 1.6 million deaths in 2021 [1]. Patients on treatment for TB are at higher risk for mental disorders as they have various triggers and shared risk factors including stigma associated with TB and HIV in case of co-infection, overcrowding, poor nutrition, smoking, alcohol use, lower income and isolation in case of multi-drug resistant tuberculosis [2–5]. Physical weakness caused by TB leads to decreased work productivity, absence from work and later job insecurity and financial instability that could be precursors or triggers of mental illness[6, 7]. Additionally, certain anti-tuberculosis drugs like isoniazid and ethambutol have side effects that include mood fluctuation, psychosis, seizures, mental confusion, dizziness and disorientation [8] and whether this relates to eventual psychiatric morbidity is yet to be ascertained.

Research on the prevalence of mental illness among patients on treatment for TB has mostly looked at depression and anxiety with a range of 35–84% for depression and 36–65% for anxiety in Ethiopia, Pakistan, India, Cameroon and South Africa [9–13]. A study from Kenya, found a prevalence of depression at 31% among 51 TB patients [14]. Studies in Zambia, Thailand and India of patients on treatment for TB found a range of 20% -30% for alcohol use disorder [15–18] while substance use disorder ranged from 3%-39% in Pakistan, Peru and Ethiopia [19–21]. Prevalence of mental illness varies depending on region and screening tool used but is higher when compared to that in the general population [22–24]. Few studies have explored psychiatric morbidity as a whole in the region and specifically in Kenya. As we aim for multisectoral collaboration to combat the TB epidemic, and as TB care teams work on holistic patient-centered care, the psychological distress and associated mental illness related to TB, cannot be ignored as it may burden the patient and affect treatment outcomes.

The presence of mental illness in TB delays health-seeking, affects drug adherence, increases cost of treatment, prolongs disease duration, lowers quality of life, and increases mortality [2, 3]. The potential for mental illness to impair adherence to TB medication increases risk of TB transmission and the development of multidrug resistance [25] and as such is a threat to public health. Mental illness affecting compliance to both anti-TB medication and anti-psychotics would lead to relapse of mental illness and further re-infection with TB [7, 25]. Knowing the prevalence, and type of patients at higher risk of mental illness among patients on treatment for TB in high TB burden countries, is crucial in directing policy to integrate mental health and TB care. Within this context, the study sought to determine the prevalence of psychiatric illness among patients on treatment for TB in Kenya. The researchers also sought to establish socio-demographic and clinical determinants, associated with psychiatric morbidity among patients on treatment for TB.

## Methods

### Study design

This was a cross-sectional study conducted between March 2019 and March 2020.

### Study setting

The study was done at the TB clinic of Moi Teaching and Referral Hospital (MTRH)—the second largest public referral hospital in Kenya. The hospital, located in the western part of the country has a wide catchment population of approximately 24 million and serves patients from both urban and rural setups. Kenya had an estimated TB incidence of 133,000 in 2021 and is ranked among the high TB and TB/HIV burden countries by the World Health Organization [1]. The TB incidence was 146,000 in 2018 prior to this study [26] and 136,000 in 2019 [27].

### Study population

All adult patients enrolled for TB treatment at the TB clinic at MTRH were eligible for recruitment into the study. On average from the TB clinic records, 400 patients were seen at the clinic every month with about 50–80 of them being newly-diagnosed with TB. Others were patients who had come for revisits and drug refill. This data was obtained in August, 2018 at the time of proposal development and was the monthly average for the preceding seven months.

### Sample size

Sample size was derived from Fishers formula where *p* was the estimated proportion of TB patients with mental illness of 31% (19), *q* = 1 –*p* (i.e. 0.69), resulting in a sample size of 329 participants. Assuming a 10% non-response the sample size was increased to a minimum of 362 respondents.

### Eligibility criteria

All adult patients with TB enrolled at the MTRH TB clinic were included. Patients who were physically severely ill or in distress or hospitalized were excluded from the study.

### Data collection

Recruitment of study participants and data collection were done simultaneously between 1^st^ August, 2019 and 28^th^ February, 2020. Interviewer -administered questionnaires were used. The first questionnaire covered socio-demographics (age, gender, marital status, level of education, occupation) and other clinical and TB characteristics including history of cigarette smoking or alcohol use, type of TB, patient type, comorbid medical illness, phase of TB treatment (initiation or continuation), prior diagnosis of psychiatric illness and family history of mental illness. The second questionnaire was the Mini International Neuropsychiatric Interview (MINI) based on Version 7 of Diagnostic and Statistical Manual 5^th^ edition (DSM-5) [28]. MINI has sixteen modules spanning a wide range of mental illness including major depressive disorder, suicidality, bipolar disorder, post-traumatic stress disorder, alcohol and substance use disorders, psychotic disorder, eating disorders, generalized anxiety disorder and antisocial personality disorder. Its validity and reliability have been assessed across multiple settings with good results [28, 29]. It has been used previously in Kenya after adaptation and adoption [30–32]. Every third patient who met eligibility criteria was approached and if they gave consent they were interviewed. This was repeated until the sample size was achieved. To get the interval of every 3^rd^ patient, we divided the average of 1200 patients expected in 3 months by the sample size 1200/367 = 3.26. This was rounded to every 3^rd^ patient to approximate the total expected patients in the duration. The data collection however took 6 months because after the initial 3 months most of the patients were revisits who had already been interviewed and hence a smaller pool to draw from.

### Analysis

The data was cleaned and analyzed using Stata v.14 (*StataCorp College Station*, *TX*, *USA*). Recategorization was carried out for some variables like age, smoking, alcohol use and the psychiatric illness. Discrete variables were summarized as frequencies and percentages and displayed as tables while continuous variables were represented with median and interquartile ranges (IQR) and categorized during further analyses.

The main outcome was presence of any psychiatric illness(es) and these were presented with their 95% confidence intervals (CI). Associations between the outcomes and the socio-demographic and clinical characteristics were assessed using Pearson’s χ2 with the strengths of association presented as odds ratio (OR) with their 95% CI. Multivariable logistic regression was used to identify factors independently associated with the main outcomes. All the socio-demographic and clinical exposure variables were included in the multivariable models unless there was collinearity e.g., HIV, diabetes and hypertension which are related with ‘any comorbidity’. The level of significance was set at *P* < 0.05.

### Ethical considerations

Ethical approval and permission were obtained from the Moi University/ Moi Teaching and Referral Hospital Institutional Research and Ethics Committee (IREC), **FAN: IREC 3276** and MTRH management respectively. Participation was entirely voluntary and all enrolled participants gave voluntary written informed consent. Anonymity was preserved by de-identification of data. Authors did not have access to information that could identify individual participants during or after data collection as only study numbers were used. Patients assessed to have serious mental health illnesses, including suicidality were referred to the mental health unit of the hospital.

## Results

### Socio-demographic and clinical characteristics of study participants

Of the 367 patients interviewed, majority (n = 225, 61%) were males with an overall median age of 33 years (IQR: 25–45). Slightly more than half of the patients (52%) were aged between 18–34 years. The proportion married (n = 155) was comparable to those single (n = 153) at 42% each, while 43 of the respondents (11.7%) were either divorced or separated. Majority (n = 354, 96%) of the participants had at least a primary level of education. In fact, almost a third (n = 105, 28%) had attained a tertiary level of education. Half (n = 184) of the patients worked in the informal sector. Half (n = 183) of the patients reported previous or current use of alcohol. Sociodemographic characteristics are shown in Table 1.

**Table 1:** Socio-demographic and clinical characteristics of patients on treatment for tuberculosis.

*Characteristic*	*Sub- category*	*n*	*(%)*
*Sex*	Male	225	(61.3%)
	Female	142	(38.7%)
*Age, years, median (IQR)*		33	(25–45)
	18 -34yrs	190	(51.8%)
	35 -49yrs	116	(31.6%)
	50 -64yrs	48	(13.1%)
	65+yrs	13	(3.5%)
*Marital status*	Single	153	(41.7%)
	Married	155	(42.2%)
	Divorced /separated	43	(11.7%)
	Widowed	16	(4.4%)
*Education*	No formal education	13	(3.8%)
	Primary	117	(32.1%)
	Secondary	129	(35.4%)
	Tertiary	105	(28.9%)
*Occupation*	Formal	52	(14.2%)
	Informal	184	(50.1%)
	Unemployed	72	(19.6%)
	Student	59	(16.1%)
*Smoking*	Never	274	(74.6%)
	Previously	77	(21%)
	Current	16	(4.4%)
*Alcohol use*	Never	183	(49.8%)
	Previously	157	(42.8%)
	Current	27	(7.4%)
*Patient type*	Newly diagnosed with TB	310	(84.5)
	Previously treated	57	(15.5)
*TB type*	PTB Bacteriologically confirmed	252	(68.7)
	PTB clinically diagnosed	32	(8.7)
	Extra-pulmonary TB	83	(22.6)
*Treatment phase*	Initiation	201	(54.8)
	Continuation	166	(45.2)
*Comorbidities*	Any comorbidity	146	(39.8)
	HIV	110	(30)
	Hypertension	17	(4.6)
	Diabetes	18	(4.9)
	Others	16	(4.3)
*Psychiatric diagnosis*	Never	358	(97.6)
	Previously	6	(1.6)
	Current	3	(0.8)
*Psychiatric Family history*	Yes	40	(10.9)
	No	327	(89.1)

TB–tuberculosis; PTB–pulmonary TB

Most of the patients interviewed (n = 310, 85%) were new (taking anti-TBs for the first time) while slightly more than half (n = 201, 55%) were in the initiation phase of treatment (first two months of treatment and on a regimen of 4 drugs). Most of the patients (n = 284, 77%) had pulmonary type TB, of which 89% was bacteriologically confirmed (sputum positive). Of the 83 patients with extrapulmonary TB, 8 had TB meningitis. Two-fifths (n = 146) of the respondents had at least one comorbidity, of which the commonest was HIV at 30% followed by diabetes (4.9%) and hypertension (4.6%). Other comorbidities included asthma, anaemia, renal disease, and cardiac disease. As regards mental illness, 358 (97.6%) of the respondents had never had a psychiatric diagnosis before while 40 (11%) had a family history of psychiatric illness. The clinical characteristics are seen in Table 1.

### Prevalence of psychiatric illnesses

Of the respondents, 48.5% (n = 178) had at least one psychiatric disorder (95% CI: 43.3–53.8). The most common psychiatric disorder among patients on treatment for TB was alcohol use disorder (AUD) at a prevalence of 30.3%. Major depressive disorder had a prevalence of 23.4% followed by substance use disorder at 12.8% and suicidality at 8.2%. Other psychiatric illnesses that were less common added up to 14.6% as in Table 2.

**Table 2:** Prevalence of specific psychiatric disorders.

*Psychiatric disorder*	*n (%)*	*(95% CI)*
*Alcohol use disorder*	111 (30.3%)	(25.6–35.2)
*Major depressive disorder*	86 (23.4%)	(19.2–28.1)
*Substance use disorder*	47 (12.8%)	(9.6–16.7)
*Suicidality*	30 (8.2%)	(5.6–11.5)
*Suicide behaviour disorder*	9 (2.5%)	(1.1–4.6)
*Mood disorder with psychotic features*	9 (2.5%)	(1.1–4.6)
*Antisocial personality disorder*	7 (1.9%)	(0.8–3.9)
*Psychotic disorder*	6 (1.6%)	(0.6–3.5)
*Panic disorder*	5 (1.4%)	(0.4–3.2)
*Obsessive compulsive disorder*	4 (1.1%)	(0.3–2.8)
*Bipolar disorder*	4 (1.1%)	(0.3–2.8)
*Generalized anxiety disorder*	3 (0.8%)	(0.2–2.4)
*Post-traumatic stress disorder*	2 (0.5%)	(0.1–2.0)
*Manic episode*	1 (0.3%)	(0.0–1.5)
*Hypomanic episode*	1 (0.3%)	(0.0–1.5)
*Agoraphobia*	1 (0.3%)	(0.0–1.5)
*Social anxiety disorder*	1 (0.3%)	(0.0–1.5)

### Determinants associated with ‘any psychiatric illness’

Of the 178 respondents with psychiatric illness, males were almost twice as likely to have any psychiatric illness compared to females (aOR 1.92, 95% CI 1.03–3.56). Patients aged 65 years and above had 88% reduced odds of any psychiatric illness as compared to those 18–34 years old (aOR 0.12, 95% CI 0.02–0.62). Being separated or divorced had almost seven times increased odds (aOR 6.86, 95% CI 2.03–23.2) of being associated with any psychiatric illness compared to the married. History of alcohol use was associated with three times the likelihood of psychiatric morbidity (aOR 3.2, 95% CI 1.68–6.08), while being previously treated for TB (aOR 2.76, 95% CI 1.26–6.04) and family history of psychiatric illness (aOR 2.40, 95% CI 1.00–5.76) was associated with more than double the likelihood. Having extra-pulmonary TB was associated with half the odds of any psychiatric illness (aOR 0.51, 95% CI 0.27–0.98). Other factors like education, occupation, treatment phase and presence of comorbidities were not significantly associated with any psychiatric morbidity as shown in **Table 3**.

**Table 3:** Determinants associated with ‘any psychiatric illnesses’.

*Characteristic*		*Yes*		*No*		*Bivariate*		*Multivariate*		
	Sub-category	n	(%)	n	(%)	cOR	p-value	aOR	95% CI	p-value
*Gender*	Female	47	(33.1)	95	(66.9)	1		1		
	Male	131	(58.2)	94	(41.8)	2.82	<0.001	**1.92**	**1.03–3.56**	**0.039**
*Age (yrs)*	18–34	73	(38.4)	117	(61.6)	1		1		
	35–49	72	(62.1)	44	(37.9)	2.62	<0.001	0.86	0.43–1.71	0.66
	50–64	30	(62.5)	18	(37.5)	2.67	<0.001	1.14	0.44–2.96	0.78
	65+	3	(23.1)	10	(76.9)	0.48	0.28	**0.12**	**0.02–0.62**	**0.011**
*Education*	No formal education	7	(53.9)	6	(46.1)	1		1		
	Primary	71	(60.7)	46	(39.3)	1.32	0.63	0.60	0.14–2.65	0.51
	Secondary	66	(51.2)	63	(48.8)	0.90	0.85	0.68	0.15–3.00	0.61
	Tertiary	31	(29.5)	74	(70.5)	0.36	0.09	0.30	0.06–1.44	0.13
*Occupation*	Formal	24	(46.1)	28	(53.9)	1		1		
	Informal	102	(55.4)	82	(44.6)	1.45	0.24	0.76	0.34–1.73	0.52
	Unemployed	40	(55.6)	32	(44.4)	1.46	0.30	1.35	0.53–3.45	0.53
	Student	12	(20.3)	47	(79.7)	0.30	0.01	0.49	0.16–1.46	0.20
*Marital status*	Married	78	(50.3)	77	(49.7)	1		1		
	Single	55	(36)	98	(64)	0.55	0.01	0.96	0.50–1.87	0.91
	Separated /divorced	39	(90.7)	4	(9.3)	9.61	<0.001	**6.86**	**2.03–23.2**	**0.002**
	Widowed	6	(37.5)	10	(62.5)	0.59	0.33	0.67	0.18–2.50	0.56
*Comorbidities*	Without	94	(42.5)	127	(57.5)	1		1		
	With	84	(57.5)	62	(42.5)	1.83	0.01	1.41	0.79–2.53	0.25
*HIV status*	No	113	(44)	144	(56)	1		1		
	Yes	65	(59.1)	45	(40.9)	1.84	0.01			
*Smoking*	Never	105	(38.3)	169	(61.7)	1		1		
	Yes	73	(78.5)	20	(21.5)	5.87	<0.001	1.51	0.72–3.19	0.28
*Alcohol use*	Never	49	(26.8)	134	(73.2)	1		1		
	Yes	129	(70.1)	55	(29.9)	6.41	<0.001	**3.20**	**1.68–6.08**	**<0.0001**
*Patient type*	New	136	(43.9)	174	(56.1)	1		1		
	Previously treated	42	(73.7)	15	(26.3)	3.58	<0.001	**2.76**	**1.26–6.04**	**0.01**
*Type of TB*	PTB bacteriological	132	(52.4)	120	(47.6)	1		1		
	PTB clinically diagnosed	16	(50)	16	(50)	0.91	0.80	0.62	0.24–1.62	0.33
	Extra-PTB	30	(36.1)	53	(63.9)	0.51	0.01	**0.51**	**0.27–0.98**	**0.04**
*Treatment phase*	Initiation	92	(45.8)	109	(54.2)	1		1		
	Continuation	86	(51.8)	80	(48.2)	1.27	0.25	1.20	0.71–2.02	0.50
*Family psychiatric history*	No	152	(46.5)	175	(53.5)	1		1		
	Yes	26	(65)	14	(35)	2.14	0.03	**2.40**	**1.00–5.76**	**0.05**

PTB–pulmonary TB; Extra-PTB–extrapulmonary TB

### Determinants associated with specific psychiatric disorders

Being separated or divorced (aOR 2.44, 95% CI 1.06–5.62) or having been previously treated for TB (aOR 2.03, 95% CI 1.00–4.1) was associated with increased odds of major depressive disorder while age 65 years and above was associated with reduced odds (aOR 0.09, 95% CI 0.01–0.89) (**Table 4**).

**Table 4:** Characteristics of TB patients with major depressive disorder.

*Characteristic*		*Yes*		*No*		*Bivariate*		*Multivariate*		
	Sub-category	n	(%)	n	(%)	cOR	p-value	aOR	95% CI	p-value
*Gender*	Female	38	(26.8)	104	(73.2)	1		1		
	Male	47	(20.9)	178	(79.1)	0.72	0.19	0.86	0.45–1.66	0.66
*Age (yrs)*	18–34	40	(21.0)	150	(79.0)	1		1		
	35–49	33	(28.4)	83	(71.6)	1.49	0.14	0.77	0.38–1.56	0.47
	50–64	11	(22.9)	37	(77.1)	1.11	0.78	0.56	0.21–1.49	0.24
	65+	1	(7.7)	12	(92.3)	0.31	0.27	**0.09**	**0.01–0.89**	**0.04**
*Education*	No formal education	5	(38.5)	8	(61.5)	1		1		
	Primary	34	(29.1)	83	(70.9)	0.66	0.49	0.45	0.11–1.95	0.29
	Secondary	31	(24.0)	98	(76.0)	0.51	0.26	0.42	0.10–1.85	0.24
	Tertiary	14	(13.3)	91	(86.7)	0.25	0.03	0.24	0.05–1.17	0.08
*Occupation*	Formal	8	(15.4)	44	(84.6)	1		1		
	Informal	42	(22.8)	142	(77.2)	1.63	0.25	1.01	0.40–2.57	0.98
	Unemployed	29	(40.3)	43	(59.7)	3.71	0.004	2.51	0.93–6.80	0.07
	Student	6	(10.2)	53	(89.8)	0.62	0.41	0.52	0.15–1.87	0.32
*Marital status*	Married	31	(20.0)	124	(80.0)	1		1		
	Single	30	(19.6)	123	(80.4)	0.98	0.93	1.02	0.51–2.06	0.95
	Separated /divorced	21	(48.8)	22	(51.2)	3.82	<0.001	**2.44**	**1.06–5.62**	**0.04**
	Widowed	3	(18.8)	13	(81.2)	0.92	0.91	0.95	0.22–4.04	0.94
*Comorbidities*	Without	43	(19.5)	178	(80.5)	1		1		
	With	42	(28.8)	104	(71.2)	1.67	0.04	1.46	0.80–2.66	0.21
*Smoking*	Never	61	(22.3)	213	(77.7)	1		1		
	Yes	24	(25.8)	69	(74.2)	1.21	0.48	0.95	0.43–2.06	0.89
*Alcohol use*	Never	40	(21.9)	143	(78.1)	1		1		
	Yes	45	(24.5)	139	(75.5)	1.15	0.56	0.92	0.45–1.87	0.81
*Patient type*	New	65	(21.0)	245	(79.0)	1		1		
	Previously treated	20	(35.1)	37	(64.9)	2.04	0.02	**2.03**	**1.00–4.10**	**0.05**
*Type of TB*	PTB bacteriological	57	(22.6)	195	(77.4)	1		1		
	PTB clinically diagnosed	12	(37.5)	20	(62.5)	2.05	0.07	1.80	0.72–4.51	0.21
	Extra-PTB	16	(19.3)	67	(80.7)	0.82	0.52	0.91	0.46–1.82	0.80
*Treatment phase*	Initiation	49	(24.4)	152	(75.6)	1		1		
	Continuation	36	(21.7)	130	(78.3)	0.86	0.54	0.88	0.51–1.53	0.65
*Family psychiatric history*	No	70	(21.4)	257	(78.6)	1		1		
	Yes	15	(37.5)	25	(62.5)	2.20	0.03	1.78	0.81–3.91	0.15

PTB–pulmonary TB; Extra-PTB–extrapulmonary TB

Being male (aOR 7.98, 95% CI 3.5–18.2), separated or divorced (aOR 3.51, 95% CI 1.3–9.65) or having history of smoking (aOR 5.03, 95% CI 2.5–10.2) was associated with increased odds of alcohol use disorder while being a student (aOR 0.02, 95% CI 0.0–0.25) and having clinically diagnosed TB (aOR 0.10, 95% CI 0.03–0.4) were associated with decreased odds (**Table 5**).

**Table 5:** Characteristics of TB patients with alcohol use disorder.

*Characteristic*		*Yes*		*No*		*Bivariate*		*Multivariate*		
	Sub-category	n	(%)	n	(%)	cOR	p-value	aOR	95%CI	p- value
*Gender*	Female	12	(8.4)	130	(91.6)	1		1		
	Male	99	(44)	126	(56)	8.5	<0.001	**7.98**	**3.5–18.2**	**<0.001**
*Age (yrs)*	18–34	37	(19.5)	153	(80.5)	1		1		
	35–49	51	(44)	65	(56)	3.24	<0.001	0.87	0.39–1.9	0.73
	50–64	21	(43.8)	27	(56.2)	3.21	0.001	0.83	0.3–2.3	0.73
	65+	2	(15.4)	11	(84.6)	0.75	0.71	0.26	0.04–2.0	0.20
*Education*	No formal education	3	(23.1)	10	(76.9)	1		1		
	Primary	50	(42.7)	67	(57.3)	2.49	0.18	1.09	0.22–5.4	0.91
	Secondary	40	(31)	89	(67)	1.50	0.55	1.10	0.22–5.5	0.90
	Tertiary	16	(15.2)	89	(84.8)	0.60	0.47	0.32	0.06–1.8	0.06
*Occupation*	Formal	19	(36.5)	33	(63.5)	1		1		
	Informal	70	(38)	114	(62)	1.07	0.84	0.42	0.2–1.04	0.06
	Unemployed	21	(29.2)	51	(70.8)	0.72	0.39	0.53	0.18–1.5	0.24
	Student	1	(1.7)	58	(98.3)	0.03	0.001	**0.02**	**0.0–0.25**	**0.002**
*Marital status*	Married	55	(35.5)	100	(64.5)	1		**1**		
	Single	26	(17)	127	(83)	0.37	<0.001	0.94	0.42–2.1	0.89
	Sep /divorced	26	(60.5)	17	(39.5)	2.78	0.004	**3.51**	**1.3–9.65**	**0.02**
	Widowed	4	(25)	12	(75)	0.61	0.41	0.98	0.21–4.4	0.98
*Comorbidities*	Without	59	(26.7)	162	(73.3)	1		1		
	With	52	(35.6)	94	(64.4)	1.52	0.07	1.39	0.72–2.7	0.32
*HIV status*	No	68	(26.5)	189	(73.5)	1		1		
	Yes	43	(39.1)	67	(60.9)	1.78	0.02			
*Smoking*	Never	48	(17.5)	226	(82.5)	1		1		
	Yes	63	(67.7)	30	(32.3)	9.89	<0.001	**5.03**	**2.5–10.2**	**<0.001**
*Patient type*	New	81	(26.1)	229	(73.9)	1		1		
	Previously treated	30	(52.6)	27	(47.4)	3.14	<0.001	2.16	0.96–4.8	0.06
*Type of TB*	PTB bacteriological confirmed	87	(34.5)	165	(65.5)	1		1		
	PTB clinically diagnosed	5	(15.6)	27	(84.4)	0.35	0.04	**0.10**	**0.03–0.4**	**0.001**
	Extra-PTB	19	(22.9)	64	(77.1)	0.56	0.05	0.65	0.31–1.4	0.26
*Treatment phase*	Initiation	57	(28.4)	144	(71.6)	1		1		
	Continuation	54	(32.5)	112	(67.5)	1.22	0.39	0.85	0.46–1.6	0.61
*Family psychiatric history*	No	97	(29.7)	230	(70.3)	1		1		
	Yes	14	(35)	26	(65)	1.28	0.49	0.79	0.31–2.0	0.62

PTB–pulmonary TB; Extra-PTB–extrapulmonary TB

Being male (aOR 16.5, 95% CI 1.9–145.2), having a history of smoking (aOR 3.57, 95% CI 1.41–9.03) or alcohol use (aOR 7.69, 95% CI 1.42–41.5) or family history of psychiatric illness (aOR 3.97, 95% CI 1.32–12.0) were statistically associated with substance use disorder (**Table 6**).

**Table 6:** Characteristics of TB patients with substance use disorder.

*Characteristic*		*Yes*		*No*		*Bivariate*		*Multivariate*		
	Sub-category	n	(%)	n	(%)	cOR	p-value	aOR	95%CI	p-value
*Gender*	Female	1	(0.7)	141	(99.3)	1		1		
	Male	46	(20.4)	179	(79.6)	36.2	0.00	**16.5**	**1.9–145.2**	**0.01**
*Age (yrs)*	18–34	16	(8.4)	174	(91.6)					
	35–49	22	(19)	94	(81)	2.55	0.00	0.79	0.27–2.32	0.66
	50–64	9	(18.8)	39	(81.2)	2.51	0.04	1.14	0.29–4.45	0.85
	65+	0	(0)	13	(100)	-		-		
*Education*	No formal education	1	(7.7)	12	(92.3)	1		1		
	Primary	25	(21.4)	92	(78.6)	3.26	0.28	1.23	0.81–18.7	0.88
	Secondary	15	(11.6)	114	(88.4)	1.58	0.67	0.78	0.05–12.0	0.05
	Tertiary	4	(3.8)	101	(96.2)	0.48	0.52	0.22	0.01–4.41	0.33
*Occupation*	Formal	5	(9.6)	47	(90.4)	1		1		
	Informal	33	(17.9)	151	(82.1)	2.05	0.16	0.81	0.24–2.78	0.74
	Unemployed	7	(9.7)	65	(90.3)	1.01	0.98	0.62	0.13–2.96	0.55
	Student	2	(3.4)	57	(96.6)	0.33	0.2	0.71	0.09–5.84	0.75
*Marital status*	Married	19	(12.3)	136	(87.7)	1		**1**		
	Single	13	(8.5)	140	(91.5)	0.66	0.28	2.12	0.66–6.88	0.21
	Sep /divorced	13	(30.2)	30	(69.8)	3.1	0.00	2.38	0.82–6.87	0.11
	Widowed	2	(12.5)	14	(87.5)	1.02	0.98	2.17	0.27–17.5	0.47
*Comorbidities*	Without	28	(12.7)	193	(87.3)	1		1		
	With	19	(13)	127	(87)	1.03	0.92	1.03	0.43–2.44	0.95
Smoking	Never	11	(4)	263	(96)	1		1		
	Yes	36	(38.7)	57	(61.3)	15.1	0.00	**3.57**	**1.41–9.03**	**0.01**
*Alcohol use*	Never	2	(1.1)	181	(98.9)	1		1		
	Yes	45	(24.5)	139	(75.5)	29.3	0.00	**7.69**	**1.42–41.5**	**0.02**
*Patient type*	New	34	(11)	276	(89)	1		1		
	Previously treated	13	(22.8)	44	(77.2)	2.4	0.02	0.90	0.33–2.43	0.84
*Type of TB*	PTB bacteriological confirmed	35	(13.9)	217	(86.1)	1		1		
	PTB clinically diagnosed	5	(15.6)	27	(84.4)	1.15	0.79	1.03	0.25–4.2	0.97
	Extra-PTB	7	(8.4)	76	(91.6)	0.57	0.20	1.04	0.36–2.99	0.94
*Treatment phase*	Initiation	25	(12.4)	176	(87.6)	1		1		
	Continuation	22	(13.2)	144	(86.8)	1.08	0.82	0.89	0.38–2.05	0.78
*Family psychiatric history*	No	36	(11)	291	(89)	1		1		
	Yes	11	(27.5)	29	(72.5)	3.07	0.00	**3.97**	**1.32–12.0**	**0.01**

PTB–pulmonary TB; Extra-PTB–extrapulmonary TB

Having other medical comorbidities like hypertension, diabetes and HIV among others was fourfold associated with suicidality (aOR 4.2, 95% CI 1.57–11.3) after adjustments.

## Discussion

### Prevalence of psychiatric morbidity

This study found that about half of the patients on treatment for TB (48.5%) had at least one mental illness, almost all of which was prior undiagnosed (97.6%). The most common disorders were alcohol use disorder (in one-third of the patients), depression (in one-in-four patients) while substance use disorder and suicidality were each prevalent in about one-in-ten patients. Generalized anxiety disorder and post-traumatic disorder were among the other less common disorders with both combined affecting one-in-a-hundred patients.

The 48.5% prevalence of any mental illness is higher compared to findings of a study in Pakistan with a rate of 31%, Nigeria at 32% and a pooled rate of 34% in a meta-analysis [19, 33, 34]. It is however lower than 82% rate in South Africa [35]. Prevalence of mental illness among patients on treatment for TB is anticipated to be higher due to shared risk factors including alcohol use, smoking, overcrowding, stigma, association with HIV, psychosocial and economic stressors or due to compromised immunity and neglected self-care associated with mental illness [25, 36]. Vulnerability, marginalization, stigma, and discrimination are often faced by people affected by TB and these could predispose to mental illness.

The low rate of a prior diagnosis of mental illness showed that there is a major treatment gap between diagnosed and undiagnosed mental illness. Similar findings were reported in another Kenyan study where of the 42% of subjects with symptoms of mental illness, only 4% had any psychiatric illness documented [31]. Health seeking for mental illness is usually affected by the associated stigma and the non-medical explanations given to mental illness. Additionally, there is lack of screening at primary health settings given that there is no diagnostic test for mental illness but rather clinical assessment or use of screening tools.

The assessed prevalence of alcohol use disorder is similar to a pooled average of 30% in a meta-analysis of 28 studies in tuberculosis patients [15], but double that previously reported for the general Kenyan population at 13.6% [37]. There’s a strong association between heavy alcohol use and TB as alcohol weakens the immune system by interfering with the integrity and microbiomes of the gastrointestinal tract, decreasing and damaging the epithelial cells and by disruption of the ciliary function of the upper airways thus ultimately increasing the risk of TB [38]. Heavy alcohol use has been linked to higher reinfection rate, poor treatment compliance and development of drug resistant TB [15]. Also people with chronic alcohol use often have poor nutritional status predisposing to activation of latent TB [3].

Prevalence of depression was lower than that in other studies with a range from 31% - 54% [9, 13, 14, 25, 39–41], likely because of the different tools used (screening versus diagnostic). Nevertheless, many studies have consistently reported higher prevalence of depression among patients with TB as compared to the general population [2, 42], and could be attributed to the psychological stressors reported by the patients including stigma, association with HIV, drug-burden especially if co-infected, long duration of treatment, lost or reduced revenue, fear of infecting people close to them, isolation from their families and increased alcohol consumption [5, 36, 43].

Prevalence of substance use was 12.8% in this study and this was a combination of all non-alcohol substances including khat/miraa, bhang, tobacco, ‘kuber’ and glue. This rate was comparable to that in a Kenyan survey with a prevalence of 13.4% [23]. Prevalence of suicidality was at 8.2% for suicidal ideation and 2.5% for suicidal attempt/ behavior. This was comparable to a study in South Africa where 9% reported suicidal ideation while 3.1% had previous suicide attempts [18]. A different study in Ethiopia had higher rates of 17.3% for suicidal ideation and 7.5% for suicide attempts [44].

### Determinants associated with psychiatric morbidity

The odds of psychiatric morbidity were increased in males, those who were separated or divorced, those with history of alcohol use, smoking, medical comorbidities, or previous history of TB and those who reported a family history of mental illness.

Males were more likely to have ‘any’ psychiatric illness or alcohol and substance use disorders, similar to findings of a meta-analysis [15] and several other studies in Africa and Asia [16–18, 45]. Culturally, social drinking among men is more tolerated compared to the same in females while it could be the start or gateway to alcohol or substance use disorders. Males, those with history of alcohol use and family psychiatric history had higher odds of substance use disorder. Those with a history of cigarette smoking even though not currently smoking still had higher odds of having substance use disorder. The gender and alcohol use association were replicated in a different study in Ethiopia where males were seven times and those with history of alcohol use were twice as likely to have substance use disorder.

Conversely, females are reported to have higher rates of depression but not alcohol or substance use disorders [11, 40, 46]. Gender differences could be as a result of males having more of externalizing symptoms as opposed to females who would present with more of internalizing symptoms [47].

This study reported lower rates of ‘any psychiatric illness’ and depression in those aged 65+. This finding of decreased rates of depression in the older age was different from other studies. A study in Ethiopia reported that for every 14 years increase in age, the risk of having depression increased by 19% [48] and another study in Nigeria reported that depression was significantly more prevalent in older patients with tuberculosis compared with their family members [5]. The difference could be due to the lower numbers in this age group given that their total number was 13, only three had any psychiatric illness and only 1 of the 13 had depression. Thus, with the lower numbers chance findings may not be ruled out.

This study found that being separated or divorced was associated with increased odds of any psychiatric illness, depression and alcohol use disorder, which were similar to other studies [10, 16, 41, 44]. Van Rensburg *et al*. in a systematic review of 100 articles also reported similar results [7]. A meta-analysis, however, reported that respondents who were single had higher odds of alcohol use disorder [15]. Those who are single, separated or divorced may lack good social support and may be at higher risk of maladaptive coping including alcohol use while on the other hand addiction may lead to change in marital status [28].

Having other medical comorbidities like hypertension, diabetes and HIV among others was fourfold associated with suicidality in this study. This was similar to studies in Ethiopia and South Africa that showed that having more than one other chronic illness and being HIV positive were positive predictors for suicidal ideation and suicide attempt [18, 44].

Other studies have similarly reported increased odds of psychiatric morbidity in TB patients who were smokers and tobacco users [17, 41], those with familial history of mental illness [11, 49], been previously treated for TB [5, 15, 38, 45, 50, 51], or with other comorbidities [8, 11, 43, 44, 52]. Substance use disorders and TB share common risk factors, including undernutrition, HIV infection, concomitant alcohol use and smoking [53]. Smoking is also considered a gateway drug to other drugs with shared risk factors for both smoking and alcohol use including genetic predisposition and family history. Studies have also reported genetic predisposition of anxiety, alcohol use disorder and depression [12, 22, 54, 55]. However, few studies have attempted to investigate the relationship of family history and mental illness among patients on treatment for TB in our setting.

The relationship between previous treatment and mental illness could be bi-directional as patients with uncontrolled mental illness could have poor adherence to anti-TB medication thus leading to retreatment as treatment interrupters or loss to follow-up. Further, because of their living conditions they could have re-infection of TB and need retreatment even after cure or treatment completion [3]. On the other hand, retreatment of TB can be a stressor given the type of drugs, long duration of treatment, drug burden and antecedent stigma associated with HIV thus predisposing to mental illness especially depression or alcohol use as a coping mechanism, albeit maladaptive.

### Limitations of the study

The main strength of this study is that the sample was socio-demographically diverse, having been drawn from a population at the second largest TB treatment program in Kenya and probably in East Africa. Secondly, the study employed a diagnostic tool (MINI) that has been validated and adapted for use in this setting. Thirdly the study is generalizable to patients on treatment for TB in similar settings based on sampling method and sample size being representative. It can, however, not be generalized to a primary care setting as this was a tertiary hospital and as such the sample may be different in a community setting.

The cross-sectional design was a limitation as causal relationships between the compared variables cannot be inferred. Much as TB could lead to or increase the risk of mental illness, this study could not establish the proportional increase in size. This thus remains an area of further research. However, the cross-sectional design was useful in bringing out the prevalence of psychiatric morbidity among patients on treatment for tuberculosis in Kenya.

Another limitation was social desirability bias due to self -reporting nature of the interviewer-administered questionnaire. Some of the sections like alcohol and substance use and suicidal ideation may attract socially acceptable responses. This would consequently cause an under-reporting of some symptoms and as such true prevalence may even be higher than reported. To mitigate against this, the interviewer employed principles of reassurance, non-judgmental attitude, and assurance of confidentiality.

Lastly, residence was not included in the socio-demographics questionnaire and considering that it impacts on socio-economics, it would additionally influence psychological and emotional well-being that would affect mental health. This was not included in this study and can be an area of research. Also, the study results found no association of females and depression which was unexpected. Further research would be needed to explain why this was different in this population.

## Conclusions

In conclusion, this study found a high prevalence of psychiatric morbidity with most common disorders being alcohol use disorder, depression, substance use disorder and suicidality. Factors associated with increased odds of having ‘any psychiatric morbidity’ were being male, being separated or divorced, history of alcohol use or smoking, family history of psychiatric illness, having medical comorbidities and being previously treated for TB.

We thus recommend for integrated and bi-directional screening, diagnosis, referral and management of comorbid TB and mental illness, akin to the TB-HIV collaborative efforts. Longitudinal studies are recommended to show causal associations and to follow up outcomes of patients with TB and psychiatric comorbidities.

## Supporting information

S1 Data (CSV)
